# Alternatives to Rapid Sequence Intubation: Contemporary Airway Management with Ketamine

**DOI:** 10.5811/westjem.2019.4.42753

**Published:** 2019-04-26

**Authors:** Andrew H. Merelman, Michael C. Perlmutter, Reuben J. Strayer

**Affiliations:** * Rocky Vista University College of Osteopathic Medicine, Parker, Colorado; † University of Minnesota Medical School, Minneapolis, Minnesota; ‡ North Memorial Health Ambulance and AirCare, Brooklyn Center, Minnesota; § Maimonides Medical Center, Department of Emergency Medicine, Brooklyn, New York

## Abstract

Endotracheal intubation (ETI) is a high-risk procedure commonly performed in emergency medicine, critical care, and the prehospital setting. Traditional rapid sequence intubation (RSI), the simultaneous administration of an induction agent and muscle relaxant, is more likely to harm patients who do not allow appropriate preparation and preoxygenation, have concerning airway anatomy, or severe hypoxia, acidemia, or hypotension. Ketamine, a dissociative anesthetic, can be used to facilitate two alternatives to RSI to augment airway safety in these scenarios: delayed sequence intubation – the use of ketamine to allow airway preparation and preoxygenation in the agitated patient; and ketamine-only breathing intubation, in which ketamine is used without a paralytic to facilitate ETI as the patient continues to breathe spontaneously. Ketamine may also provide hemodynamic benefits during standard RSI and is a valuable agent for post-intubation analgesia and sedation. When RSI is not an optimal airway management strategy, ketamine’s unique pharmacology can be harnessed to facilitate alternative approaches that may increase patient safety.

## INTRODUCTION

Airway management and endotracheal intubation (ETI) are life-saving interventions frequently performed in emergency medicine (EM), critical care, and prehospital medicine. Despite its prevalence, ETI is associated with considerable patient morbidity and mortality, and is considered the riskiest commonly-performed procedure in acute care.^1^,^2^ Rapid sequence intubation (RSI), which uses the simultaneous administration of an induction agent and paralytic, is the most common method of facilitating ETI. Traditional RSI, however, is burdened by the crucial risks of hypoxia and acidosis should ETI and assisted ventilation fail, as well as hypotension and hypoperfusion caused by the abrupt transition from negative-pressure to positive-pressure ventilation.^3^

Ketamine, a dissociative anesthetic classically used to facilitate painful procedures in non-intubated patients, has unique properties that offer patient-safety advantages over traditional RSI induction agents. These properties can be leveraged in novel ways to permit alternative pharmacologic approaches that mitigate RSI risks. Because dissociative doses of ketamine disconnect the patient from external stimuli while brainstem function remains intact, painful or distressing procedures such as ETI can be performed on the unaware, dissociated patient while cardiorespiratory tone is preserved or augmented.^4^ This allows the provider to modify traditional RSI in ways that address the most important RSI risks.

Two alternatives to RSI have emerged that harness ketamine’s unique pharmacology to improve airway management safety in specific clinical scenarios: delayed sequence intubation (DSI) – the use of ketamine to allow airway preparation and preoxygenation in the agitated patient; and ketamine-only breathing intubation (KOBI), which uses ketamine without a paralytic to facilitate ETI as the patient continues to breathe spontaneously. In this narrative review we discuss these techniques, neither of which at present is supported by clear evidence.

In conventional RSI, ketamine has become a preferred induction agent because of its relative hemodynamic stability (compared to propofol, midazolam, and thiopental) and long duration of action (compared to propofol and etomidate). Additionally, ketamine provides analgesia, amnesia, and sedation in a single agent, making it well-suited for post-intubation sedation.

## DISCUSSION

### Ketamine to Facilitate Preoxygenation in the Uncooperative Patient: Delayed Sequence Intubation

Many patients who require intubation do not allow appropriate preparation for intubation–most importantly preoxygenation–due to agitation, which may be from hypoxia, intoxication, or a variety of other cooperation-impairing conditions. This dangerous scenario is particularly common when clinicians attempt to use face mask noninvasive ventilation (continuous positive airway pressure or bilevel positive airway pressure) for preoxygenation. Performing RSI on a hypoxemic patient significantly increases morbidity and mortality,^5^–^7^ and an adequate period of preoxygenation is the most important strategy in prolonging the period of RSI-induced apnea during which ETI is safely completed.^8^ The patient ripping off his or her face mask is often the patient most in need of optimal oxygenation techniques and the most likely to be harmed by suboptimal preparation and preoxygenation.

DSI uses a dissociative dose of ketamine to render the patient unconscious while airway, breathing, and circulatory tone are maintained so that preparation and preoxygenation can proceed to completion. The original DSI study demonstrated the technique for use in preoxygenation or for pre-intubation nasogastric tube placement in upper gastrointestinal hemorrhage.^9^ DSI starts with dissociative-dose ketamine: 1–2 milligrams per kilogram (mg/kg) intravenously (IV) or 4–6 mg/kg intramuscularly. Once the patient is dissociated and unconscious, providers have achieved control of a dangerous, uncontrolled situation and can proceed with preoxygenation and other preparations such as placement of adequate vascular access, assembly of necessary equipment and personnel, and initiation of therapies targeting the patient’s underlying condition. Once preparation and preoxygenation are complete, a usual paralytic dose is administered and laryngoscopy proceeds, as in RSI.

In the original case series of 62 emergency department (ED) and intensive care unit (ICU) patients, oxygen saturation improved from 90% to 99% on average with DSI, and two asthma patients improved sufficiently following ketamine administration that they no longer required intubation. More recently, a prehospital package of care including DSI, apneic oxygenation, video laryngoscopy, and proper positioning reduced adverse events when compared to patients undergoing standard RSI.^10^ Additional publications have demonstrated the effectiveness of DSI when undertaken by flight paramedics^11^ and suggested its utility in critically ill pediatric patients.^12^,^13^ When ketamine is pushed IV, as a quick bolus, it may cause a brief period of apnea that is usually self-limited but is undesired and can typically be avoided by administering ketamine over 30–60 seconds, which may require dilution.^4^,^14^ Providers should be prepared to proceed immediately with paralytic administration and laryngoscopy if dangerous hypoventilation or airway compromise occurs during the period of dissociation.

### Ketamine to Facilitate ETI in the Spontaneously Breathing Patient: Ketamine-only Breathing Intubation

The use of ketamine monotherapy–without a paralytic–to facilitate intubation is an emerging technique that offers pivotal benefits over RSI in specific circumstances. Its effectiveness has been demonstrated in field and military environments but has not yet been widely adopted in EM.^15^,^16^ Performing ETI using only induction agents has a long history in prehospital medicine and is generally referred to as medication-assisted intubation (MAI), where deep sedation is induced using a combination of fentanyl and midazolam or diazepam, followed by laryngoscopy. MAI has performed poorly when studied and is associated with failed intubation attempts, vomiting, hypoxia, hypotension, cardiac arrest, and under-sedation.^17^–^19^ Midazolam has been shown to substantially diminish airway muscle activity.^20^ Dissociative-dose ketamine, however, reliably renders the patient impervious to and amnestic of ETI (or any other painful stimuli) while airway reflexes, respiration, and blood pressure are typically maintained.^4^ Ketamine-only breathing intubation (KOBI) is the use of dissociative-dose ketamine to facilitate intubation in the spontaneously breathing patient, with or without the addition of topical anesthesia. This technique has been described as ketamine-assisted intubation, ketamine-facilitated intubation, ketamine-only intubation, ketamine-supported intubation, and dissociated awake intubation.^21^–^23^ Etomidate may also be used without a paralytic to facilitate a breathing airway technique.^18^ Procedural sedation experience suggests that etomidate is more likely to cause myoclonus or muscle rigidity, however, compared to ketamine.^24^,^25^

Despite a growing interest in KOBI, there is a lack of published experience with the procedure; the description and recommendations herein are based on expert opinion and intended to provide a framework for safety and efficacy. KOBI allows ETI to be performed while the patient continues to breathe, in the fashion of what is often called an awake intubation; however, the term *awake* applies poorly to the unconscious, dissociated patient; strategies employed with the goal of preserving spontaneous respirations are better referred to as breathing techniques. KOBI is primarily useful in airways that are known or predicted to be anatomically difficult (e.g., anatomic factors such as obesity, limited neck mobility, or oropharyngeal tumor that may hinder the operator from visualizing the glottis or passing the endotracheal tube through the vocal cords). These patients are typically managed in elective anesthesia settings using thorough local anesthesia and flexible endoscopy (e.g., fiberoptic bronchoscopy). However, this truly awake technique requires time and patient cooperation, as well as skills and equipment that may not be available to emergency or prehospital providers. KOBI may provide a similar degree of safety to traditional awake flexible endoscopic intubation, does not require additional time or a cooperative patient, and uses pharmacology and laryngoscopy techniques familiar to all acute care airway operators.

The second group of patients who may benefit from continuous breathing throughout airway management have signs of physiologic difficulty, insofar as they are predicted to clinically deteriorate during or immediately after intubation – in particular, patients who may not tolerate even a brief period of apnea. The most common example is patients who have a high oxygenation deficit, which is evident when oxygen saturation cannot be improved above 95% on high-flow supplemental oxygen using either a face mask, or non-invasive ventilation. These patients, who may have pneumonia, acute respiratory distress syndrome, or other forms of structural lung disease, are at high risk to dangerously desaturate immediately after breathing slows and ceases during RSI; using a breathing technique to facilitate ETI may, therefore, have important safety benefits. Because ketamine-dissociated patients are sedated they may develop reduced minute ventilation. But in patients where reduced minute ventilation is significantly advantageous compared to apnea, using a breathing technique to facilitate ETI may have important safety benefits compared to paralysis. Very hyperdynamic patients with high heart rate and blood pressure (e.g., severe alcohol withdrawal, thyroid storm) are a less-recognized group that desaturate quickly from their high oxygen extraction and may similarly benefit from a breathing technique during airway management.

Profoundly acidemic patients (e.g., diabetic ketoacidosis, toxic alcohol ingestion, lactic acidosis) have a high ventilation deficit and require very high minute ventilation. Because they are also at high risk for peri-intubation decompensation, they may benefit from allowing spontaneous respiration as an alternative to RSI-induced apnea. Serum pH is not monitored continuously as is oxygen saturation; thus, these patients are not recognized as deteriorating and instead develop “sudden” cardiac arrest during or after airway management.

Another category of patient who may benefit from KOBI is the patient with dangerous hypotension and a high perfusion deficit, whose predisposition to deteriorate during or after intubation is mitigated by an induction that has minimal impact on hemodynamics. Apnea and the transition from negative- to positive-pressure ventilation reduces venous return and, in physiologically marginal patients, may precipitate circulatory collapse.^26^ Using a breathing technique during intubation followed by gentle and gradually augmented pressure support afterward may improve outcomes in critically ill patients requiring airway management.

Whether or not a breathing technique such as KOBI is used, all physiologically marginal patients should be explicitly evaluated for their potential to develop critical hypoperfusion during and after ETI; point-of-care sonography to assess cardiac contractility and volume status may have particular value in this context.^27^ Patients who are judged to be a high physiologic risk should be resuscitated prior to intubation to the extent possible by maximizing therapies directed at the underlying pathophysiological insults such as crystalloid or blood, antibiotics, and vasopressor support.

Dissociated patients may have muscle rigidity, including a clenched jaw, which can typically be mitigated using small doses of a conventional sedative such as midazolam or propofol, or a sub-induction dose of etomidate; however, these adjuncts may also cause hypoventilation or apnea. Furthermore, patients intubated using any breathing technique, including KOBI, may develop vomiting, laryngospasm, and apnea,^28^ for which the operator must be prepared. Compared to breathing techniques, the use of a paralytic during ETI provides the optimal view of the glottis and abolishes airway reflexes such as coughing and gagging that may hinder glottic exposure and tube placement. For these reasons, a fast-acting paralytic (rocuronium or succinylcholine) must be readily available in syringe when performing KOBI to address laryngospasm, muscle rigidity, or inadequate view due to muscle tone. Until and unless alternative methods for preventing or treating ketamine-related muscle rigidity are demonstrated, KOBI should only be undertaken if a neuromuscular blocking agent is available.

Providers may also address some of these disadvantages of intubating the spontaneously breathing patient by using a bougie or flexible endoscope, and by providing topical anesthesia to the posterior oropharynx as time, patient cooperation, and resources allow. We recommend the application of 4% lidocaine using a flexible-tipped atomization device, just ahead of the gradually advanced laryngoscope, to blunt sensation in the soft palate, periglottic tissues, and vocal cords. If glottic view is adequate but airway reflexes or vocal cord movement prevent successful tube or bougie placement, administration of a paralytic as laryngoscopy is maintained is an appropriate breathing technique modification, especially when the initial concern was anatomic difficulty.

The relative benefits and risks of RSI vs a breathing technique should be considered for every intubation procedure: the more features of an anatomically or physiologically difficult airway, the more time available, and the lower the risk of vomiting, the greater the potential benefit of using a breathing technique. Appropriate preparation for any emergency airway procedure includes material readiness with all relevant airway equipment at the bedside including a paralytic agent drawn up in a syringe, as well as cognitive readiness through formulating and verbalizing a comprehensive airway management plan prior to commencing the procedure.

### Ketamine in Traditional Rapid Sequence Intubation

In standard RSI, when apnea caused by the induction agent is not a concern (as apnea is intentionally caused by the paralytic agent), ketamine has advantages over other agents: primarily its positive or neutral hemodynamic effect in most patients.^29^ Peri-intubation hypotension correlates with mortality,^5^,^6^,^30^ and ketamine is therefore favored in hemodynamically compromised patients. As a weak sympathomimetic, ketamine is more likely to maintain tissue perfusion during and after RSI, compared to fentanyl, midazolam, thiopental, and especially propofol.^29^,^31^–^33^ In patients with a high shock index, ketamine has been demonstrated to maintain blood pressure^34^ and is associated with post-intubation hypotension less frequently than other induction agents.^35^,^36^ However, ketamine, like any sedative, can cause or worsen hypotension in catecholamine-depleted patients in shock.^37^ Patients with high perfusion deficits who require ETI are therefore ideally resuscitated prior to intubation, and the induction dose of ketamine – like all induction agents – should be reduced by at least half (from 1–2 mg/kg to 0.5–1 mg/kg IV) in these cases.^38^ Profoundly hypoperfused or obtunded patients should receive even smaller doses, and the peri-arrest comatose patient may be more likely to be harmed than helped by even a small dose of an induction agent and may be reasonably intubated with a paralytic only. Ketamine should be dosed based on ideal body weight, as estimated by patient height, not actual body weight.^39^

Ketamine’s long duration of action, compared to etomidate, and especially propofol, is an important advantage in EM and prehospital medicine, as post-intubation sedation is often delayed in these environments.^40^,^41^ Ketamine is also thought to have intrinsic action as a bronchodilator and is the preferred induction agent for patients being intubated for obstructive lung disease.^42^

### Ketamine for Post-Intubation Analgesia and Sedation

Patients intubated in the ED or prehospital may receive suboptimal post-intubation analgesia and sedation,^43^–^45^ especially those who received long-acting paralytic agents and therefore do not show signs of distress. Acute care providers may find it technically difficult to administer and titrate both analgesic and sedative drips and may be reluctant to use conventional agents in hemodynamically tenuous patients. Ketamine is safe and effective for post-intubation analgosedation^46^–^48^ and has two primary benefits over alternatives: ketamine is catecholaminergic and therefore stimulating to heart rate and blood pressure, and ketamine has both analgesic and sedative properties, which allow ketamine to be used as monotherapy in the intubated patient. Use of ketamine in mechanically ventilated patients also allows dose reductions of conventional sedatives,^49^ which have been linked to prolonged ICU length of stay and delirium.^50^

Although experience is limited, ketamine seems best suited to provide analgosedation in the period immediately after intubation, when the goal is deep unconsciousness during the resuscitative phase of critical illness. During this period, ketamine may be used in dissociative doses, using a 1–2 mg/kg bolus (if ketamine was not used as the induction agent during ETI), followed by a drip rate of 1–5 mg/kg per hour, titrated to effect. Patients given subdissociative doses of ketamine are conscious and often experience psychoperceptual effects that may cause psychiatric distress; it is therefore more challenging to use ketamine as a post-intubation analgosedative when the patient has stabilized, and lighter planes of anesthesia are desired. If ketamine is used in subdissociative doses, psychiatric distress is effectively managed with conventional sedatives such as benzodiazepines, propofol, or butyrophenone neuroleptics, if needed. Particularly advantageous to chaotic emergency and prehospital environments, ketamine may be used in dissociative bolus dosing to immediately effect patient stillness and unawareness, as drips are being set up or titrated.

## CONCLUSION

The introduction of paralytics and RSI into airway management performed outside the operating room was an important advance in patient safety and in the development of prehospital and emergency medicine. Since then, the rise of video laryngoscopy has diminished the advantage of paralysis in improving the view of the glottis, and the expanded use of ketamine has revealed that dissociated patients tolerate laryngoscopy as the patient continues to breathe spontaneously. Contemporary airway operators have learned to harness the advantages of video laryngoscopy and ketamine’s unique properties to develop RSI alternatives that offer safety benefits during airway management for patients who do not allow optimal preparation or are especially likely to be harmed using a paralytic (See Figure). These strategies currently have a limited base of experience and evidence, and as with any airway management technique should be executed with planning, deliberation, and caution.

## Figures and Tables

**Figure:**
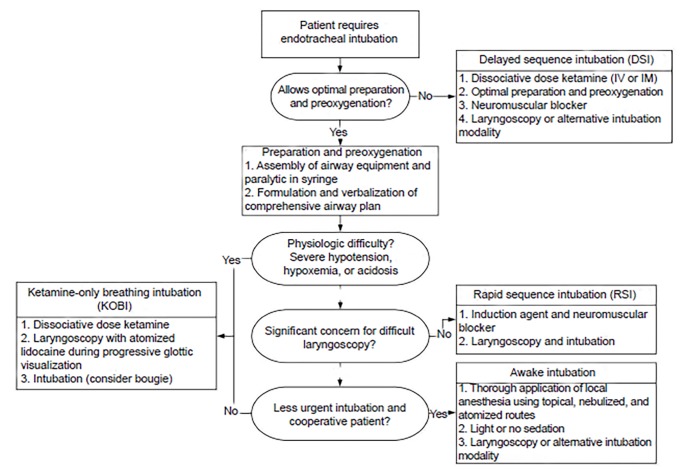
Algorithm providing general guidance for determining which is the most appropriate technique for urgent or emergent endotracheal intubation. *IV*, intravenous; *IM*, intramuscular.
